# Genome sequencing and mining expand the naturalproduct repertoire of Lysobacter

**DOI:** 10.21203/rs.3.rs-4939843/v1

**Published:** 2024-09-17

**Authors:** Jeffrey J. Bierman, Mark C. Walker

**Affiliations:** University of New Mexico; University of New Mexico

**Keywords:** Lysobacter, genome mining, natural product, plusbacin

## Abstract

**Background:**

Compounds produced by living organisms serve as an important source of inspiration for the development of pharmaceuticals. A potential source of new natural products are bacteria from a genus with species that are known to produce bioactive natural products, but are relatively understudied. *Lysobacter* is a genus of bacteria that have attracted attention as possible biocontrol agents and are known to produce antibiotic natural products. To further explore the biosynthetic potential of *Lysobacter*, we sequenced the genomes of two species and performed genome mining studies on those and publicly available genomes.

**Results:**

In this study we produced draft genome sequences for *Lysobacter firmicutimachus* and *Lysobacter yananisis*. We additionally examined 113 publicly available *Lysobacter* genomes and found that biosynthetic potential of individual species ranges broadly, with species having between 1 and nearly 20 biosynthetic gene clusters. Filtering for more complete genome assemblies and 9 or more biosynthetic gene clusters, we performed genome mining on 24 *Lysobacter* genomes. Within these genomes we identified 21 unique nonribosomal peptide, 11 unique hybrid polyketide/nonribosomal peptide, 4 unique polyketide, and 27 unique lanthipeptide biosynthetic gene clusters that produce uncharacterized compounds. Additionally, we tentatively identified the biosynthetic gene cluster in *L. rmicutimachus* responsible for producing plusbacins, which has not been previously identified.

**Conclusions:**

This study demonstrated that *Lysobacter* have a large repertoire of natural products that remain to be characterized. Additionally, we found that some *Lysobacter* species are substantially more biosynthetically gifted than others and that strains of the same species of *Lysobacter* have similar biosynthetic capacities.

## Background

Chemicals produced by living organism, also known as natural products or secondary metabolites, serve as an important source for pharmaceuticals [1]. Indeed, nearly a third of small molecule drugs in the clinic are, or are derivatives of, natural products [2]. The continued search for new pharmaceuticals, particularly antibiotics to help address the growing challenge of antibiotic-resistant bacterial infections [3, 4], necessitates further exploration of the biosynthetic capacity of nature. A potentially fruitful source of new compounds could be bacteria that are relatively understudied for their capacity to produce natural products.

*Lysobacter* is a genus of Gram-negative gammaproteobacteria that have garnered interest as biocontrol agents due to their ability to induce lysis of a number of microorganisms [5]. This ability is due to their secretion of enzymes such as chitinases [6–8], which can degrade fungal cell walls and the eggshells of nematodes, glucanases [9, 10], which can degrade fungal cell walls and contribute to antioomycete activity, and peptidases [11, 12] that contribute to broad antimicrobial activity and nematicial activity. In addition to these bioactive enzymes, *Lysobacter* species produce natural products. These natural products include nonribosomal peptides such as lysobactin [13, 14], WAP-8294A [15], tripropeptins [16], plusbacins [17], hypeptin [18], lysocins [19] and WBP-29479A1 [20], which have drawn interest due to their activity against Gram-positive pathogens such as *Staphylococcus aureus*. *Lysobacter* species produce other natural products with antimicrobial activity such as cephabacins [21, 22] and heat stable antifungal factor [23].

Beyond the bioactive compounds that have been isolated from *Lysobacter* species, genome mining studies have revealed a number of potential biosynthetic gene clusters that could encode the biosynthesis of natural products that have not yet been characterized [24, 25]. These studies focused on the genomes of *Lysobacter enzymogenes*, *Lysobacter capsici*, *Lysobacter antibioticus*, and *Lysobacter gummosus*. To gain further insight into the biosynthetic repertoire of *Lysobacter*, we sequenced the genomes of two more species, *Lysobacter firmicutimachus*, which has been reported to produce plusbacins, and *Lysobacter yananisis*. We then predicted the biosynthetic gene clusters (BGCs) in their genomes and compared those gene clusters to those in other publicly available *Lysobacter* genomes, and explored the natural product biosynthesis of *L. firmicutimachus* and *L. yananisis* by mass spectrometry.

## Methods

### *Lysobacter* strains and culture conditions

*L. yananisis* was purchased from American Type Culture Collection (ATCC) as BAA-2621 and was received as a freeze-dried cell pellet in a double vial while *L. firmicutimachus* was purchased from the German Collection of Microorganisms and Cell Cultures (DSMZ) as DSM No.: 102073 and was also received as a freeze-dried cell pellet in a double vial. Cells were reconstituted following the instructions of the provider, with sterile Tryptic Soy Broth (TSB) growth media used for initial liquid cultures and sterile Tryptic Soy Agar (TSA) plates for initial streaking and colony picking. Growth of these species took place at 30°C in aerobic conditions, with liquid cultures being shaken at 180 RPM for oxygenation. Genomic DNA was extracted from the initiated cultures (NEB Monarch Genomic DNA Purification Kit, Cat. No. T3010L) following their instructions for gram-negative cells. Universal primers (U1 F: ccagcagccgcggtaatacg, U1 R: atcggctaccttgttacgacttc) were used in PCR amplification of the 16s rRNA subunit from the gDNA samples for each species, the PCR products were then subjected to sanger sequencing and BLAST searches to confirm the identity of the bacteria. Glycerol stocks were made from the 16s rRNA confirmed samples, and subsequent cultures used in this study were initiated from these stocks that were kept at −80°C.

### Isolation of genomic DNA

For both *L. yananisis* and *L. firmicutimachus*, a single colony was picked and grown overnight in 5 mL of TSB at 30°C with shaking. These overnight cultures were used to inoculate new 5 mL TSB cultures to an OD600 of 0.05. These cultures were grown at 30°C with shaking until the OD_600 nm_ reached 0.8, when the cells were collected by centrifugation. Genomic DNA was isolated according to previously reported procedure [26]. Briefly, the cell pellet was resuspended in 5 mL of SET buffer (75 mM NaCl, 25 mM EDTA, 20 mM Tris, pH 7.5) and incubated with 2 mg of lysozyme for 1 h at 37°C. Then 2.8 mg of proteinase K and 0.6 mL of 10% SDS were added and the solution was incubated for 2 h at 55°C. 2 mL of 5 M NaCl was then added and mixed thoroughly by inversion. 5 mL of chloroform was then added and incubated at room temperature with rocking for 30 min. The organic and aqueous layers were separated by centrifugation, the aqueous layer was recovered and treated twice more with chloroform. The genomic DNA was precipitated by adding 0.6 volumes of isopropyl alcohol, spooled around a pipet tip, washed with 70% ethanol, and dissolved in a minimal amount of deionized water.

### Sequencing of Genomic DNA by Azenta Life Sciences:

Frozen genomic DNA samples were express shipped on dry ice to Azenta Life Sciences for the preparation of PacBio CLR libraries with multiplexing barcodes and subsequent PacBio Sequel sequencing. The quantity and quality of amplicon DNA were assessed and the PacBio library was prepared using SMRTbell prep kit, with bead size selection per the manufacturer’s protocol. The run design guidelines for sequencing were followed and the SMRTbell libraries were sequenced on the PacBio Sequel platform, with one BAM file being generated per SMRTcell. Generated reads were demultiplexed, and the sorted output files were returned from Azenta for genome assembly and annotation

### Genome Assembly and PGAP Annotation:

Raw read quality was assessed using LongQC v1.2.1 [27], the assembly of the reads proceeded with Canu v2.2 [28] with default settings, then the assemblies were polished with the Flye v2.9.2-b1795 [29] polishing tool. Inspector v1.0.2 [30] was used as a final check to assess assembly quality before submitting the fasta files for genome annotation by the Prokaryotic Genome Annotation Pipeline (PGAP) on NCBI [31].

### Genome mining

Genbank files for *Lysobacter* genomes were downloaded from NCBI on September 19, 2023. The percent contig N_50_ of total genome length was calculated using the accompanying data summary file. Genome assemblies with a contig N_50_ greater than 80% of the total length of the genome were submitted to antiSMASH 7.0 [32] with the default settings. Genome assemblies with nine or more biosynthetic regions were further examined. NRPS, PKS/NRPS, and PKS encoding BGCs were manually sorted based on the predicted specificity of their building block selecting domains. Lanthipeptide precursor peptides were manually identified by examining short open reading frames in the BGC for the presence of many Ser, Thr, and Cys residues in the C-terminal portion of the peptide. The sequence similarity network of lanthipeptide precursor peptides was generated using the Enzyme Function Initiative’s Enzyme Similarity Tool [33]. 16s rDNA sequences were extracted from the downloaded genbank files with a Biopython [34] script. The sequences were aligned with Muscle [35] and the phylogenetic tree was created with FastTree 2 [36] and visualized with the Interactive Tree of Life v6 [37].

### Culturing conditions for natural product screening

*L. yananisis* was streaked onto TSB-agar plate from glycerol stock, *L. firmicutimachus* was streaked onto R2A-agar plate from glycerol stock, and both were placed into a 30°C incubator to grow overnight. Fresh YPM [0.5% (w/v) yeast extract, 0.3% (w/v) peptone, 0.5% (w/v) D-mannitol, DI water][13] growth media was prepared and sterilized in the autoclave (121°C, 20 minutes), then 5 mL seed cultures for each species were inoculated from the developed plates and grown for 3.5 hours in a temperature-controlled shaker (180 RPM, 30°C). 50 mL YPM fermentation cultures in baffled culture asks were inoculated to an OD_600 nm_ of 0.05 from the seed cultures and placed into the shaker for 3 days (180 RPM, 30°C). Once grown cultures were removed from the shaker and each was poured into an individual sterile 50mL conical tube to be spun down (12,500 × g, 4°C, 30 minutes). Supernatants from each were poured into new individual sterile 50mL conical tubes, then 45 μL of formic acid (LC-MS grade) were added. Samples were vortexed, spun down (12.5 × g, 4°C, 30 minutes) to pellet any precipitate, then each sample was filtered through a PES 25mm 22-μm filter disk into an individual sterile 15 mL conical tube (12 mL for *L. yananisis*; 3.5 mL for *L. firmicutimachus*, which quickly clogged the filter disks, requiring 4 filters to get this volume). 250 μL of each were transferred into fresh epi-tubes and kept frozen before being for storage prior to being analyzed by mass spectrometry.

### Orbitrap LC-MS DDA Media Screening:

Spent media samples analyzed on a Thermo Scientific Q-Exactive Orbitrap LC-MS with an UltiMate 3000 RSLCnano UHPLC module for liquid chromatography. Frozen 250 μL culture samples were thawed, 60 μL of sample was loaded onto an Agilent C18 cleanup kit following the manufacture protocol, with the only difference being that 90% ACN with 0.1% TFA was used to elute the sample for less peptide retention. 30 μL water with 0.1% formic acid was added to the samples after clean-up. Samples were kept at 7°C in the autosampler before 1 μL injections were performed. The LC method was 120 minutes long, with a flow rate of 3 μL/min and starting mobile phase of 98% H2O with 0.1% formic acid (solvent A) and 2% Acetonitrile with 0.1% formic acid (solvent B). After eight minutes a gradient to 40% B initiated and ended at 80 minutes, followed by another gradient to 95% B by 90 minutes where it held at that ratio until 100 minutes. The mobile phase composition returned to 2% B by 105 minutes where it held until the end of the run at 120 minutes. A Thermo Scientific ES902 EASY-Spray column was used for reverse-phase separation of compounds.

The mass spectrometry settings were for a Full MS/dd-MS2 (Top N) experiment in positive mode covering 120 minutes, with a mass range of 375–4,000 Da for the full MS scans and a mass range of 200–2,000 Da for the MS/MS scans. The Top N experiment had a loop count of 5 and MSX count of 1, with a mass isolation window of 1.0 m/z and intensity threshold of 8.0e4.

## Results

### Genome sequencing

The assembled *Lysobacter firmicutimachus* genome was 5.3 Mb over 3 contigs. This genome encoded 4,615 genes, with 4,495 protein coding genes, two rRNA operons with 5s, 16s, and 23s rRNA encoding genes, and 56 tRNA encoding genes. The assembled *Lysobacter yananisis* genome was 6.2 Mb also over 3 contigs, and this genome encoded 5,126 genes with 5,003 protein coding genes, two rRNA operons with 5s, 16s and 23s encoding genes, and 61 tRNA encoding genes. Comparing these genomes to those from other *Lysobacter* species revealed that the *L. firmicutimachus* genome was most similar to *L*. sp. BMK333–48F3 with an average nucleotide identity of 91.2%, and the *L. yananisis* genome was most similar to *L. enzymogenes* B25 with an average nucleotide identity of 98.0% [38].

### Lysobacter genomes

**Figure 1:**
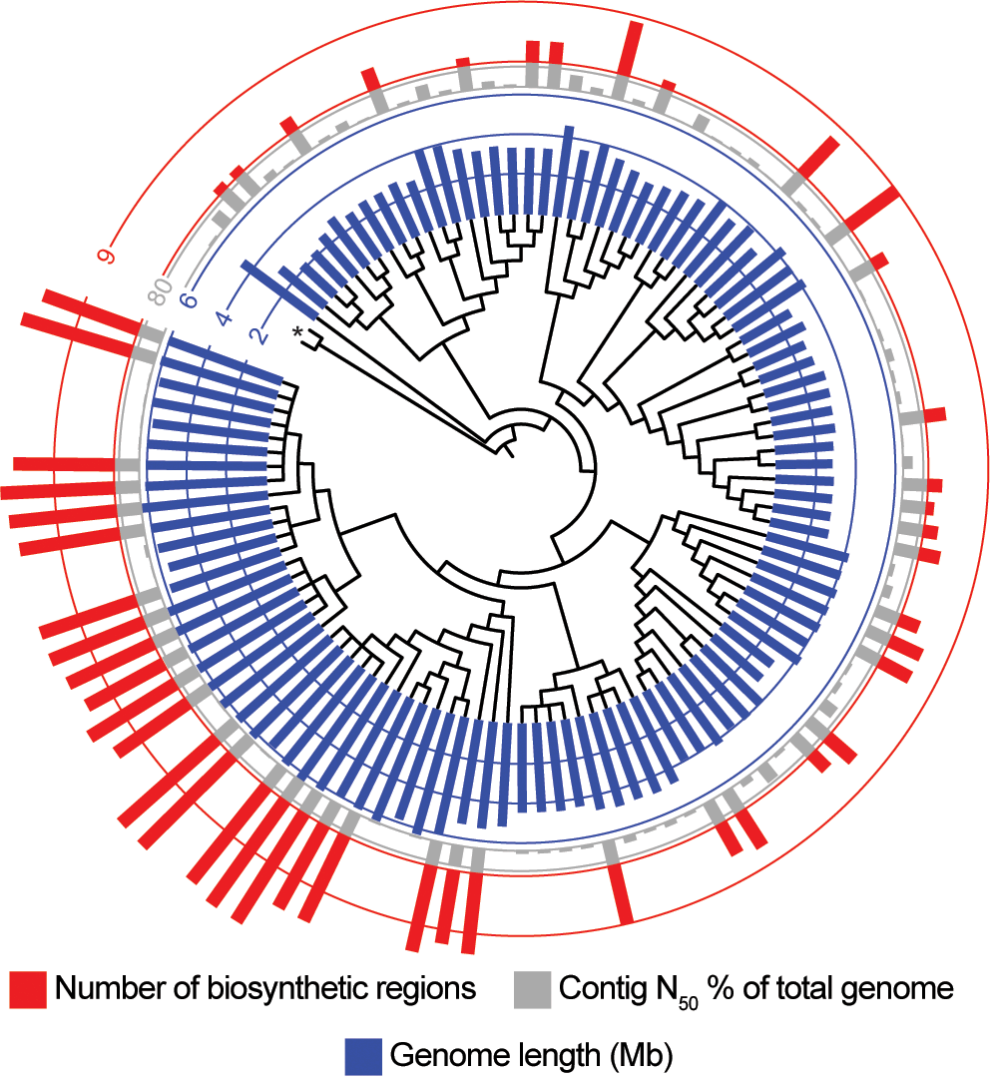
Phylogenetic tree of *Lysobacter* 16s rDNA. Phylogenetic tree constructed from the 16s rDNA sequences from *Lysobacter* genomes available on NCBI. Tracks show the length of the genome, the percent of the total genome length the N_50_ of the assembly, and the number of secondary metabolite regions identified by antiSMASH. * indicates leaves from 16s rDNA of *Escherichia coli* str K-12 substr MG1655 and *Pseudomonas fluorescens* ATCC 13525 as out groups.

113 available *Lysobacter* genomes were downloaded from NCBI (September 2023). These genomes ranged from 2.3 to 6.4 Mb in length (Fig. 1). To facilitate examination of BGCs we focused on genome assemblies where the contig N_50_ was at least 80% of the total genome length, as assemblies with shorter contigs often have BGCs split over multiple contigs, which makes analysis challenging. This filter resulted in 46 genomes. We then analyzed these genomes by antiSMASH [32] to predict the number of secondary metabolite regions present in each genome. These secondary metabolite regions may contain more than one BGC if those BGCs are located close together in the genome. This analysis revealed between 1 and 19 secondary metabolite regions per genome. We focused on the 22 genomes with at least 9 secondary metabolite regions plus those of *L. firmicutimachus* and *L. yananisis* for further analysis. These genomes were on the longer end of *Lysobacter* genomes, with none being shorter than 4.0 Mb.

### Biosynthetic gene clusters

#### Non-ribosomal peptides

Among the genomes that were examined, 108 non-ribosomal peptide biosynthetic gene clusters were identified (Figure S1). Based on predictions of the building blocks incorporated by the modules in the non-ribosomal peptide synthetases (NRPS) encoded in these BGCs, these clusters encode the production of 21 distinct natural products or very similar natural products. Of these BGCs, the products of 6 have been characterized. Among the BGCs with characterized products, the BCG for WAP-8294A is the most widely distributed, appearing in 10 genomes, including that of *L. yananisis*. The next most abundant BGCs with characterized products are the Le-pyrolopyrazines, lysobactin, and WBP-29479A1, appearing in 7, 5, and 3 genomes respectively. The BGCs for hypeptin in L. sp. K5869 and tripropeptin in *L*. sp. BMK333–48F3 did not appear in any of the other genomes we examined.

**Figure 2:**
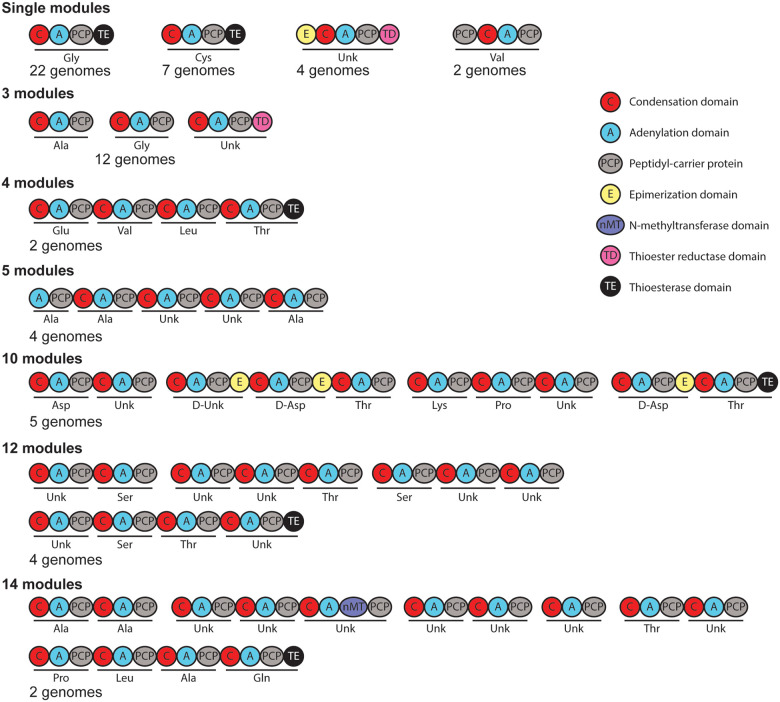
Nonribosomal peptide synthetases. Nonribosomal peptide synthetases not connected to a known natural product identified in *Lysobacter* genomes. Modules are underlined with the predicted selectivity of their adenylation domains indicated. Separate polypeptides are indicated by a space between domains.

The most common NRPS BGC without a characterized product appears 25 times in the examined genomes, with some genomes containing more than one copy of this BGC. A single NRPS module that is predicted to incorporate a glycine is encoded in this BGC (Fig. 2). Three other BGCs that encode a single NRPS module that incorporate different amino acids were also identified. The next most common BGC, in 12 genomes, encodes three modules, with each module encoded in a separate gene. Other BGCs encoding larger NRPSs with 5, 10, 12, 14, and 17 modules were found to be shared among more than one genome. None of the identified NRPS BGCs without characterized products appear to encode the production of compounds that differed from characterized nonribosomal peptides from *Lysobacter* by a few amino acid residues.

*L. firmicutimachus* has been reported to produce the plusbacins, which have been thoroughly studied through chemical synthesis [39, 40], but the biosynthetic gene cluster encoding their production has not been reported. A BGC that is a candidate for the encoding this production was identified in the *L. firmicutimachus* genome (Figure S2). The NRPS encoded in this cluster is predicted to incorporate the residues in plusbacin, except for one module which is predicted to incorporate a Ser residue where plusbacin has an Ala residue. The NRPS also contains dual epimerization/condensation domains [41] that would result in D-amino acids being installed where those residues are in plusbacin. Additionally, the cluster encodes two TauD family dioxygenases, which could be responsible for installing the hydroxyl groups of the 3-hydroxy proline and β-hydroxy aspartate residues present in the plusbacins.

#### Hybrid polyketide/non-ribosomal peptides

42 hybrid polyketide-nonribosomal peptide BGCs were identified in the genomes that were examined and these BGCs encode the production 11 distinct hybrid polyketide-nonribosomal peptide compounds. A number of polycyclic tetramate macrolactams [42], produced by hybrid PKS-NRPSs have been characterized from *Lysobacter* species. Indeed, polycyclic tetramate macrolactam BGCs were the most common hybrid PKS-NRPS clusters identified, appearing in 18 of the examined genomes. The majority of these clusters are very similar and encode three flavin dependent oxidoreductases and one NAD dependent dehydrogenase, which are involved in installing the polycyclic structure of heat stable antifungal factor [ref]. However, the clusters in L. sp. BMK333–48F3, L. sp. K5869, and *L. firmicutimachus* only encode two flavin dependent oxidoreductases potentially encoding the production of Alteramide [42]. Additionally, the BGC for lysohexaenetides [43] was identified in two genomes.

**Figure 3:**
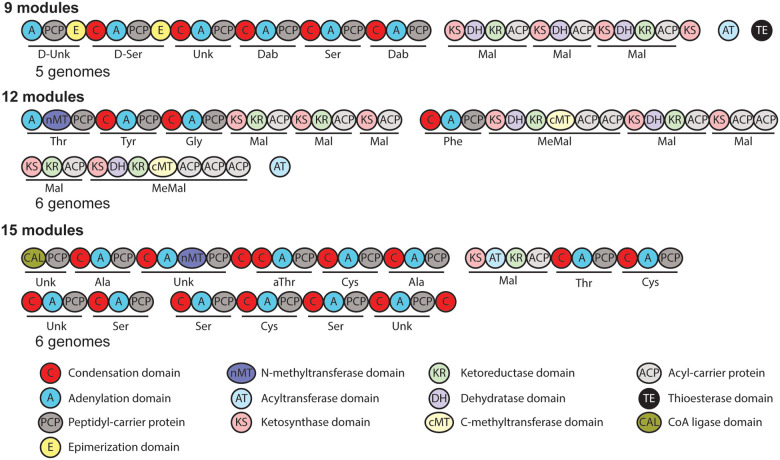
Hybrid polyketide synthase-nonribosomal peptide synthetases. Hybrid polyketide synthase-nonribosomal peptide synthetases that are not connected to known natural products identified in *Lysobacter* genomes. Modules are underlined with the predicted selectivity of their adenylation or acyltransferase domains indicated. Separate polypeptides are indicated by a space between domains.

Three hybrid PKS-NRPS BGCs with uncharacterized products in more than one genome were identified (Fig. 3). Two of the three encode trans-AT PKSs as part of the hybrid synthase. The third encodes a hybrid PKS-NRPS with a single PKS module with a cis-AT domain.

#### Polyketides

**Figure 4:**
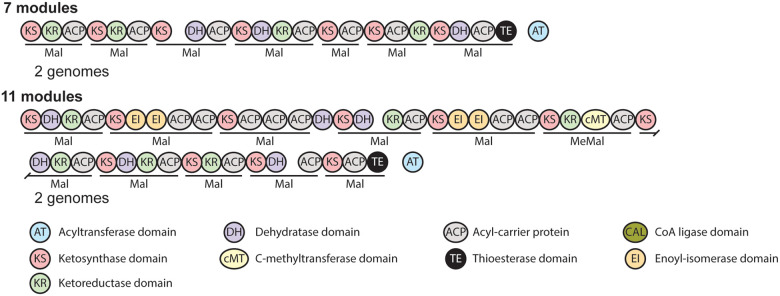
Polyketide synthases. Polyketide synthases that are not connected to known natural products identified in *Lysobacter* genomes. Modules are underlined with the predicted selectivity of their trans acyltransferase domains indicated. Separate polypeptides are indicated by a space between domains.

Six type I PKS BGCs were identified, potentially producing 4 different compounds. No type II or type III PKS BGCs were identified, despite a type II PKS being responsible for the production of Lysobacter pigment [44]. A BLAST [45] search of the examined genomes revealed a number of them likely contain Lysobacter pigment BGCs that were not identified by antiSMASH. Two of these PKS BGCs are shared between more than one genome and encode trans-AT PKSs (Fig. 4).

#### Lanthipeptides

**Figure 5:**
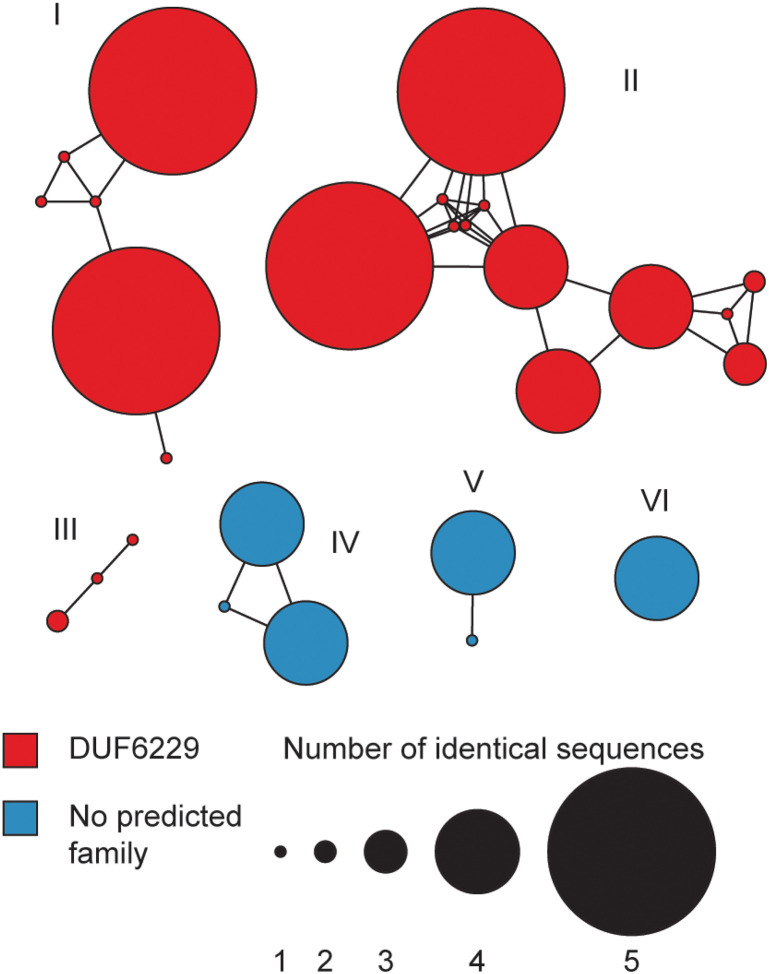
Sequence similarity network of lanthipeptide precursors. A sequence similarity network of lanthipeptide precursor peptides was constructed from those identified in *Lysobacter* genomes.

72 class II lanthipeptide BGCs were identified that encoded 27 unique precursor peptides. The majority of these precursor peptides, 21 of the 27 unique precursor peptides, are annotated as a domain of unknown function (DUF6229). A lanthipeptide with a precursor peptide belonging to DUF6229 has been reported to exhibit antibiotic activity against *K. pneumoniae* [46], however the positions of Cys, Ser, and Thr residues in the predicted core peptides identified in *Lysobacter* are different, suggesting the products could have different structures (Figure S3). Indeed, generating a sequence similarity network of the precursor peptides revealed the precursors cluster into six families (Fig. 5), each of which appear to have different macrocyclization topologies. Additionally, 8 class III lanthipeptide BGCs were identified with 3 unique precursor peptides.

#### Natural products from *L. yananisis* and *L. firmicutimachus*

**Figure 6:**
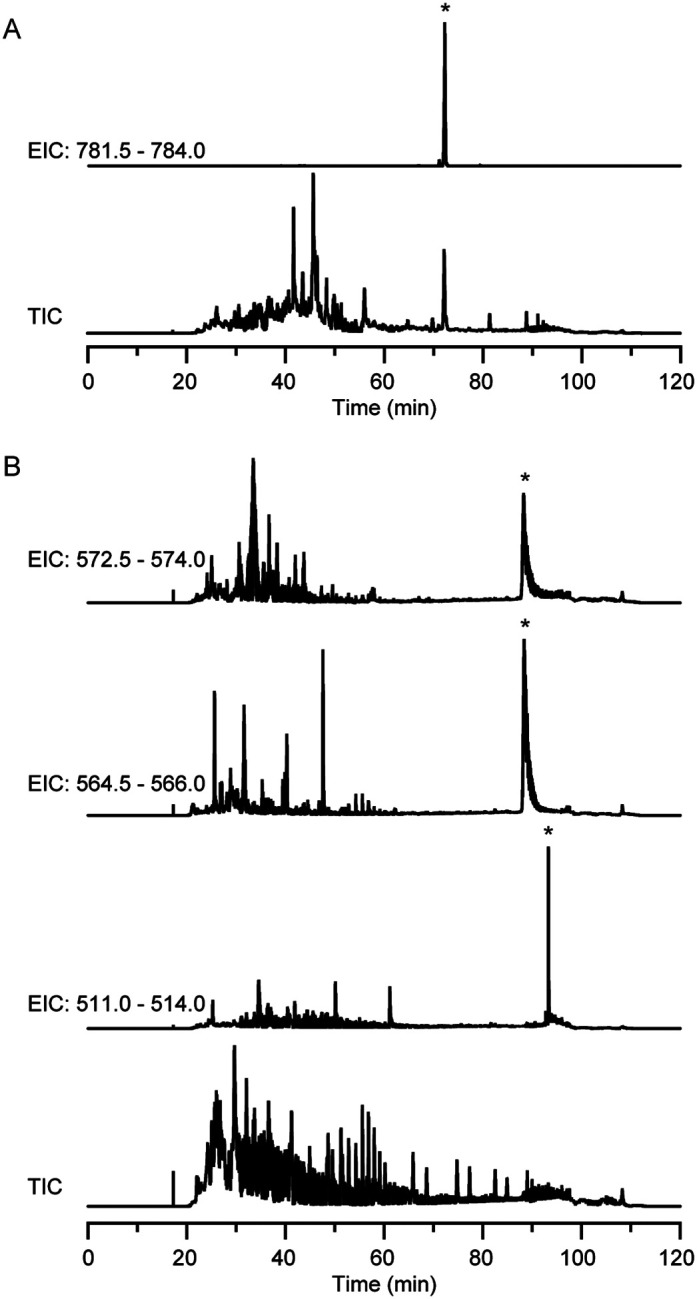
Natural products from *L. yananisis* and *L. firmicutimachus*. (A) Chromatograms from *L. yananisis*. The extracted ion chromatogram encompasses the expected m/z for the doubly charged state of WAP-8294A (781.9143). (B) Chromatograms from *L. firmicutimachus*. The extracted ion chromatograms encompass the expected m/z for doubly charged plusbacin A_2_ (572.7903), doubly charged plusbacin B_2_ (564.7928) and singly charged alteramide A or alteramide B (511.2803). * indicates the peaks with tandem mass spectra that match the expected fragmentation patterns.

To briefly explore natural products produced by *L. yananisis* and *L. fimicutemachus*, these organisms were cultured and the spent cultures were analyzed by LC/MS. Production of WAP-8294A by *L. yananisis* was observed (Fig. 6, Supplementary Fig. 4). In the *L. firmicutimachus* spent media, a signals consistent with plusbacin A_2_, plusbacin B_2_, and alteramide were detected (Fig. 6). Tandem mass spectrometry performed on the putative plusbacin A_2_ and plusbacin B_2_ was consistent with previously reported fragmentation results [47], further supporting the identification (Supplementary Fig. 5). Tandem mass spectrometry of the putative alteramide was less informative, but still consistent with previously reported results for polycyclic tetramate macrolactams (Supplementary Fig. 6) [48]. While other compounds were detected in the spent media, none of the m/z values or tandem mass spectra could readily be linked to a predicted BGC.

## Discussion

Here we have reported the genome sequence of *L. yananisis* and *L. rmicutimachus*. Comparison of these genome sequences with those of previously determined genome sequences reveal that the genome of *L. yananisis* is very similar to that of strains of *L. enzymogenes*. Indeed, the 98% average nucleotide identity between *L. yananisis* and *L. enzymogenes* B25 is above the generally accepted threshold [49] to consider two bacteria to be from the same species. It is possible that *L. yananisis* should be considered a strain of *L. enzymogenes* rather than a separate species.

Additionally, we explored the biosynthetic capacity of 46 species of *Lysobacter*. Notably, not all of the species harbored a large number of biosynthetic gene clusters. While some encoded the production of as many as 19 different natural products, others potentially encoded the production of only a single natural product. This diversity in the number of BGCs present suggest that, should further genome sequencing of *Lysobacter* be undertaken for the purpose of identifying new BGCs, prioritization of those species more closely related to the more biosynthetically gifted species may be warranted. Our analysis also revealed that different strains of the same species of *Lysobacter* generally have a similar repertoire of BGCs. In particular, strains of *L. enzymogenes*, *L. capsici*, and *L. gummosus* appear to have the potential to produce many of the same natural products.

Examination of the potential BGCs revealed *Lysobacter* species can produce nonribosomal peptides, hybrid nonribosomal peptide/polyketides, polyketides, and lanthipeptides that have not yet been characterized. However, culturing *L. yananisis* and *L. firmicutimachus* only resulted in the production of known compounds. This result highlights the challenges associated with activating cryptic BGCs under laboratory conditions, suggesting other approaches are needed to elicit production of these compounds for determination of their structure and biological activities.

## Conclusion

*Lysobacter* species harbor the capacity to produce many uncharacterized natural products.

## Data Availability

Sequence data associated with this study has been deposited at NCBI. The *L. yananisis* sample (BioSample: SAMN39658516) was assigned GenBank Accession Number (JBANDK010000001) and the *L. firmicutimachus* sample (BioSample: SAMN39658517) was assigned GenBank Accession Number (JBANDL000000000). Both biosamples were assigned to a single bioproject (BioProject: PRJNA1070720) for this work.

## Abbreviations

BGC: biosynthetic gene cluster
NRPS: nonribosomal peptide synthetase
PKS: polyketide synthase
